# Brief research report: Effects of Pinch deficiency on cartilage homeostasis in adult mice

**DOI:** 10.3389/fcell.2023.1116128

**Published:** 2023-01-19

**Authors:** Xiaohao Wu, Sixiong Lin, Rongdong Liao, Qing Yao, Lijun Lin, Xuenong Zou, Guozhi Xiao

**Affiliations:** ^1^ Department of Biochemistry, Shenzhen Key Laboratory of Cell Microenvironment, Guangdong Provincial Key Laboratory of Cell Microenvironment and Disease Research, School of Medicine, Southern University of Science and Technology, Shenzhen, China; ^2^ Guangdong Provincial Key Laboratory of Orthopaedics and Traumatology, Department of Spine Surgery, The First Affiliated Hospital of Sun Yat-sen University, Guangzhou, China; ^3^ Department of Orthopaedics, Zhujiang Hospital, Southern Medical University, Guangzhou, China

**Keywords:** Pinch, focal adhesion, articular cartilage, osteoarthritis, homeostasis

## Abstract

Pinch1 and Pinch2 are LIM domain-containing proteins with crucial functions in mediating focal adhesion formation. Our previous studies have demonstrated that Pinch1/2 expression is essential for cartilage and bone formation during skeletal development in mice. Loss of Pinch expression (Prx1^Cre^; Pinch1^flox/flox^; Pinch2^−/−^) inhibits chondrocyte proliferation and promotes chondrocyte apoptosis, resulting in severe chondrodysplasia and limb shortening. Based on these observations, we wonder if Pinch proteins have a role in adult cartilage and whether Pinch deficiency will compromise cartilage homeostasis and promote osteoarthritis (OA)-related defects in adult mice. To this end, we generated the Aggrecan^CreERT2^; Pinch1^flox/flox^; Pinch2^−/−^ mice, in which the Pinch1 gene can be inducibly deleted in aggrecan-expressing chondrocytes by tamoxifen and the Pinch2 gene is globally inactivated. Immunofluorescent staining confirmed that the expression of Pinch proteins was significantly decreased in articular cartilage in tamoxifen-treated adult Aggrecan^CreERT2^; Pinch1^flox/flox^; Pinch2^−/−^ mice. Unexpectedly, our results showed that Pinch loss did not induce marked abnormalities in articular cartilage and other joint tissues in the knee joints of either adult (10-month-old) mice or aged (17-month-old) mice. In a destabilization of the medial meniscus (DMM)-induced OA model, the surgically-induced OA lesions were comparable between Pinch-deficient mice and control mice. Given the fact that Pinch proteins are essential for chondrogenesis and cartilage formation during skeletal development, these findings suggest that Pinch expression is seemingly not indispensable for adult cartilage homeostasis in mice.

## Introduction

Pinch1 and Pinch2 are evolutionarily conserved proteins, which contain five cysteine-rich LIM domains to mediate protein-protein interactions (Tu et al., 1999). Pinch proteins interact with integrin linked kinase (ILK) and parvin to form a heterotrimeric ILK-Pinch-parvin (IPP) complex (Legate et al., 2006). The IPP complex binds to the cytoplasmic tail of extracellular matrix (ECM)-ligated integrins and links them to the actin cytoskeleton to facilitate the cell-ECM adhesion (Wickström et al., 2010). The Pinch proteins are reported to be involved in multiple fundamental cellular processes, such as focal adhesion (FA) formation, cytoskeletal organization, cell proliferation, migration, and survival (Fukuda et al., 2003; Sakai et al., 2003; Li et al., 2005; Liang et al., 2005; Chen et al., 2008; Xu et al., 2016). Global deficiency of the Pinch1 gene (Pinch1^−/−^) is lethal in mice (Liang et al., 2005), whereas global Pinch2-null (Pinch2^−/−^) mice displayed no obvious abnormalities (Stanchi et al., 2005). Results from our group have demonstrated that loss of Pinch proteins leads to severe defects in multiple organs and tissues in mice (Wang et al., 2019; Lei et al., 2020; Gao et al., 2021). For instance, Pinch deficiency in Dmp1-expressing osteocytes disturbs the balance between bone formation and resorption, leading to severe osteopenia phenotypes in mice (Wang et al., 2019). More importantly, we have demonstrated that loss of Pinch expression in mesenchymal progenitor cells (Prx1^Cre^; Pinch1^flox/flox^; Pinch2^−/−^) causes severe bone and cartilage defects during skeletal development, including small body size, limb shortening, chondrodysplasia, and reduced bone mass, in mice (Lei et al., 2020). In growth plate cartilage, loss of Pinch proteins significantly inhibits chondrocyte proliferation, enhances chondrocyte apoptosis, and disrupts chondrocyte column formation. These findings suggest that Pinch proteins play essential roles in mediating chondrogenesis and cartilage formation during skeletal development.

Based on these observations, we wonder whether Pinch proteins have a role in adult articular cartilage and whether Pinch deficiency will compromise cartilage homeostasis and promote osteoarthritis (OA)-related defects in adult mice. To this end, we generated the Aggrecan^CreERT2^; Pinch1^flox/flox^; Pinch2^−/−^ mice, in which the Pinch1 gene can be inducibly deleted in aggrecan-expressing chondrocytes by tamoxifen and the Pinch2 gene is globally inactivated. The effects of Pinch1/2 deletion on adult articular cartilage as well as other joint tissues and OA-related scores were quantitatively determined in both spontaneous and surgically-induced OA models.

## Result

### Generation of Aggrecan^CreERT2^; Pinch1^flox/flox^; Pinch2^−/−^ mice

**FIGURE 1:**
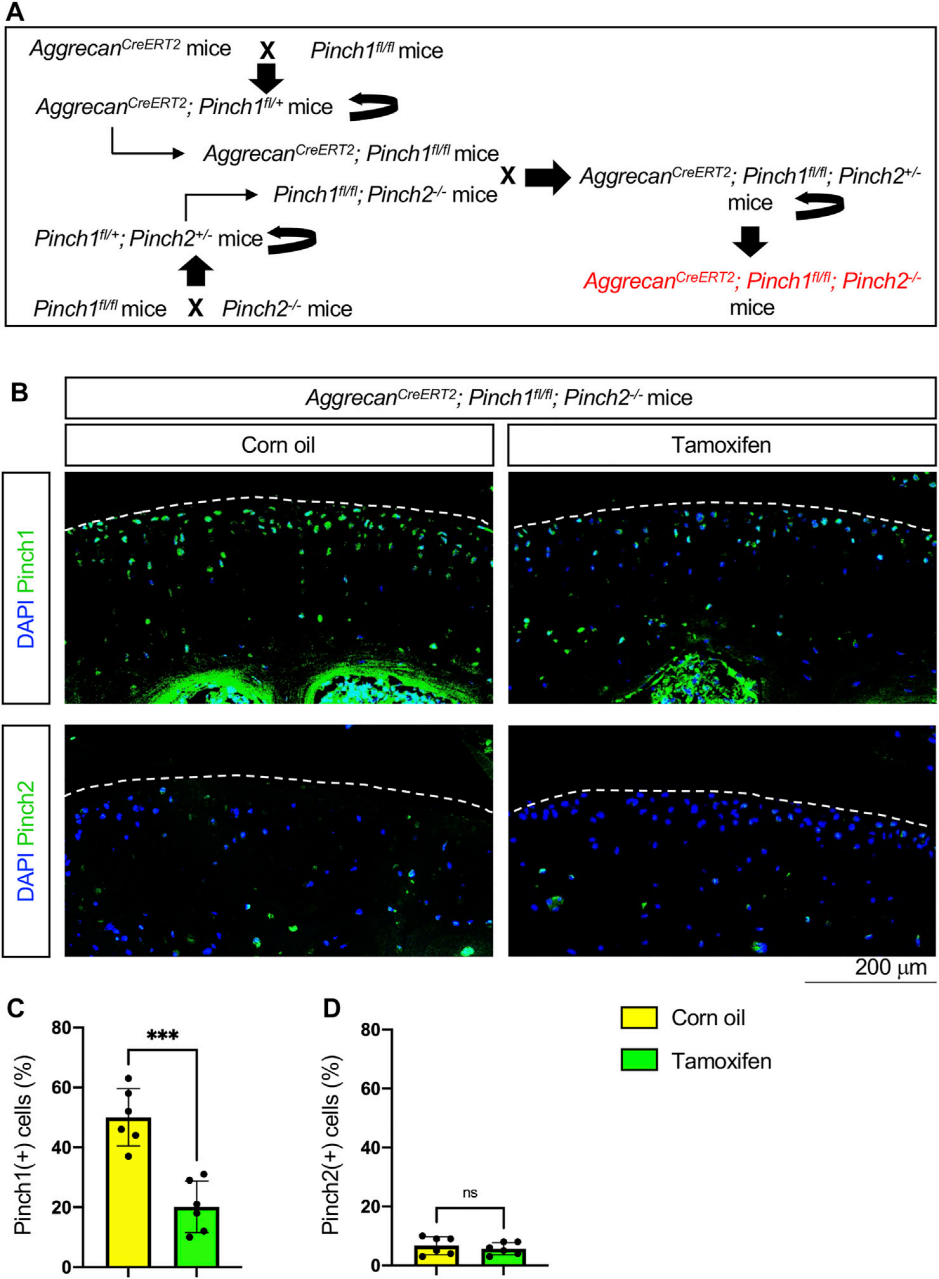
Generation of Aggrecan^CreERT2^; Pinch1^flox/flox^; Pinch2^−/−^ mice. **(A)** A schema showing the breeding of Aggrecan^CreERT2^; Pinch1^flox/flox^; Pinch2^−/−^ mice. Tamoxifen was used to induce Pinch1 deletion in aggrecan-positive chondrocytes in 2-month-old Aggrecan^CreERT2^; Pinch1^flox/flox^; Pinch2^−/−^ mice. Corn oil was used as the control treatment of tamoxifen. **(B)** Immunofluorescence staining images showing the expression of Pinch1 and Pinch2 proteins in articular cartilage of knee joint. Scale bar: 200 μm. **(C–D)** Percentages of Pinch1- and Pinch2-positive chondrocytes in tamoxifen- and corn oil-treated Aggrecan^CreERT2^; Pinch1^flox/flox^; Pinch2^−/−^ mice, respectively. Note: N = 6 mice for each group. Results are expressed as mean ± standard deviation (s.d.). ***p < 0.001; ****p < 0.0001.

To investigate whether Pinch proteins have a role in adult cartilage, we generated the Aggrecan^CreERT2^; Pinch1^flox/flox^; Pinch2^−/−^ mice by crossing the Aggrecan^CreERT2^ mice with floxed Pinch1 (Pinch1^flox/flox^) mice and Pinch2-null (Pinch2^−/−^) mice, as indicated in Figure 1A. At the age of 2 months, the male Aggrecan^CreERT2^; Pinch1^flox/flox^; Pinch2^−/−^ mice were administrated with 5 daily intraperitoneal injections of tamoxifen (100 mg/kg body weight, dissolved in corn oil) to induce Pinch1 deletion in aggrecan-expressing chondrocytes. Notably, age-matched male Aggrecan^CreERT2^; Pinch1^flox/flox^; Pinch2^−/−^ mice were treated with corn oil and served as a control group. Immunofluorescent staining analyses showed that, at 1 week after tamoxifen treatment, the expression of Pinch1 was significantly decreased in articular chondrocytes of tamoxifen-treated mice, as compared with those in corn-oil-treated controls (Figure 1B, C). Since the Pinch2 gene is globally inactivated in Aggrecan^CreERT2^; Pinch1^flox/flox^; Pinch2^−/−^ mice; as expected, the expression of Pinch2 was barely detectable in articular chondrocytes in both groups (Figure 1B, D).

### Pinch1/2 loss does not induce marked abnormalities in knee joints in adult mice

**FIGURE 2:**
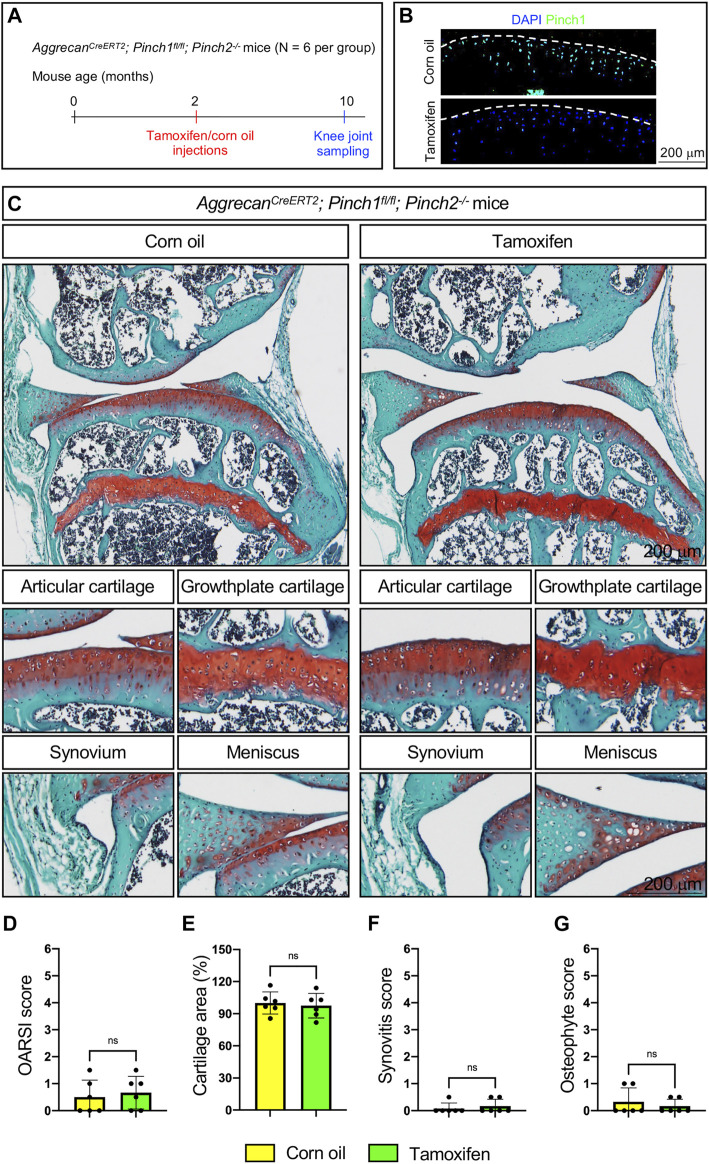
Pinch1/2 loss does not induce marked abnormalities in knee joints in adult mice. **(A)** A schema illustrating the experimental design. **(B)** Immunofluorescence staining of Pinch1 in articular cartilage. Scale bar: 200 μm. **(C)** Representative safranin O and fast green-stained images of knee joint sections from tamoxifen- or corn oil-treated Aggrecan^CreERT2^; Pinch1^flox/flox^; Pinch2^−/−^ mice at 10 months of age. Higher magnification images in lower panels are showing the areas of articular cartilage, growth-plate cartilage, synovium, and meniscus. Scale bar: 200 μm. **(D–G)** Quantitative histological analyses of OARSI (osteoarthritis research society international) score, cartilage areas, synovitis score, and osteophyte score using knee joint sections. Note: N = 6 mice for each group. Results are expressed as mean ± standard deviation (s.d.). ns: not significant.

To determine whether Pinch1/2 deficiency could induce structural abnormalities in adult articular cartilage, six 2-month-old male Aggrecan^CreERT2^; Pinch1^flox/flox^; Pinch2^−/−^ mice were treated with tamoxifen. Another six age-matched Aggrecan^CreERT2^; Pinch1^flox/flox^; Pinch2^−/−^ mice were treated with corn oil as controls. Eight months later, all mice were sacrificed, and the knee joint samples were collected (Figure 2A). Immunofluorescent staining analyses confirmed that the protein expression of Pinch1 was markedly decreased in the articular cartilage of tamoxifen-treated mice, as compared with control mice (Figure 2B). We performed safranin O and fast green staining and quantitative histological analyses to assess the joint structure and OA-related parameters, including the OARSI (osteoarthritis research society international) score, cartilage area, synovitis score, and osteophyte score. The results showed no marked difference in articular cartilage and other joint tissues, including growth plate cartilage, synovium, and meniscus (Figure 2C). Moreover, the OA-related parameters were comparable between tamoxifen- and corn oil-treated Aggrecan^CreERT2^; Pinch1^flox/flox^; Pinch2^−/−^ mice (Figures 2D–G).

### Pinch1/2 loss does not promote OA defects in aged mice

**FIGURE 3:**
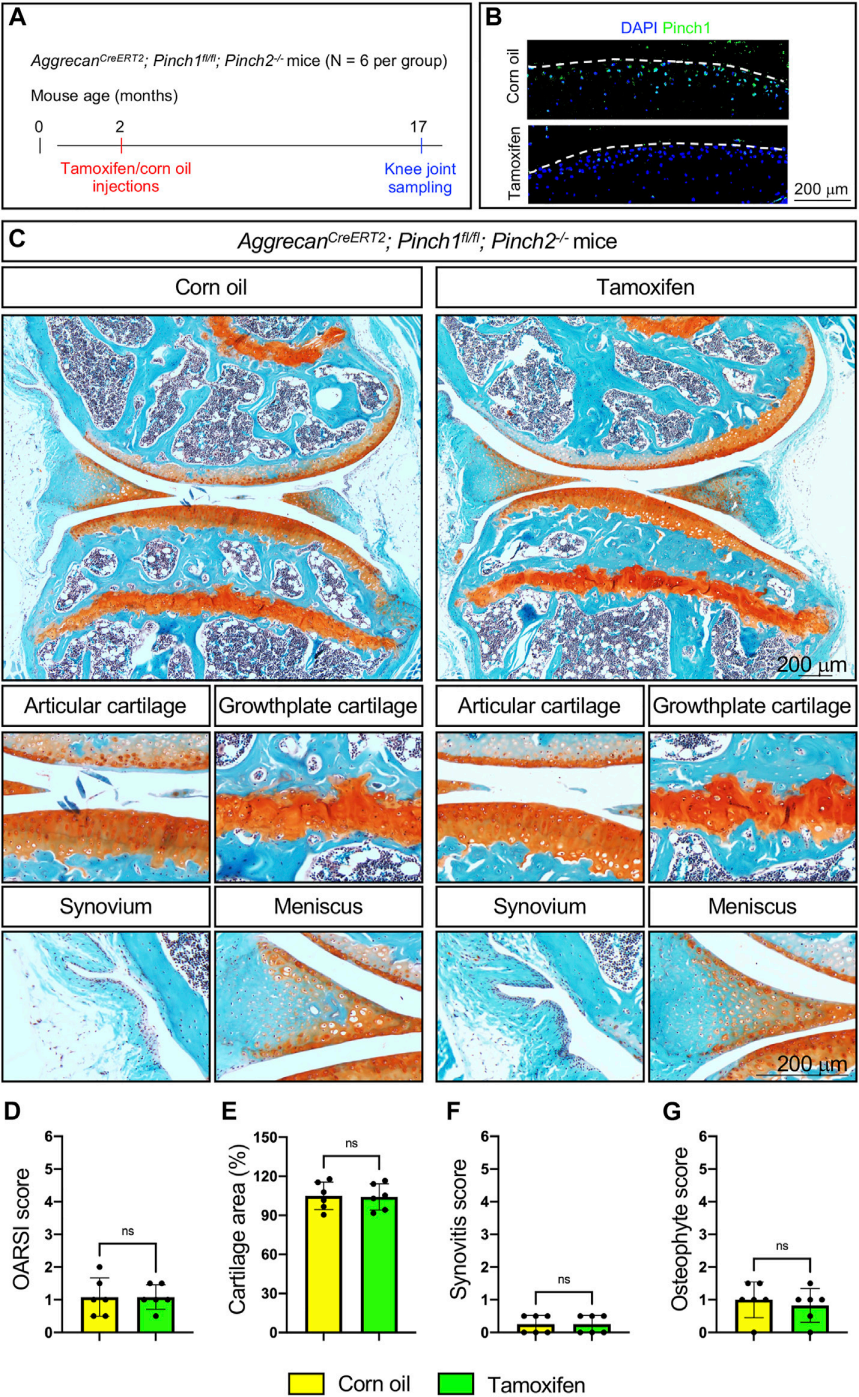
Pinch1/2 loss does not promote OA defects in aged mice.** (A)** A schema illustrating the experimental design. **(B)** Immunofluorescence staining of Pinch1 in articular cartilage. Scale bar: 200 μm. **(C)** Representative safranin O and fast green-stained images of knee joint sections from tamoxifen- or corn oil-treated Aggrecan^CreERT2^; Pinch1^flox/flox^; Pinch2^−/−^ mice at 17 months of age. Higher magnification images in lower panels are showing the areas of articular cartilage, growth-plate cartilage, synovium, and meniscus. Scale bar: 200 μm. **(D–G)** Quantitative histological analyses of OARSI score, cartilage areas, synovitis score, and osteophyte score using knee joint sections. Note: N = 6 mice for each group. Results are expressed as mean ± standard deviation (s.d.). ns: not significant.

Next, we tested if Pinch1/2 deficiency could promote spontaneous OA defects in aged mice. Briefly, six 2-month-old male Aggrecan^CreERT2^; Pinch1^flox/flox^; Pinch2^−/−^ mice were treated with tamoxifen, and another six age-matched Aggrecan^CreERT2^; Pinch1^flox/flox^; Pinch2^−/−^ mice were treated with corn oil. At the age of 17 months, all mice were sacrificed, and the knee joint samples were collected (Figure 3A). The Pinch1 expression was markedly decreased in the articular cartilage of tamoxifen-treated mice, as compared with control mice (Figure 2B). Histological analyses demonstrated no significant alterations in knee joints and OA-related parameters between tamoxifen- and corn oil-treated groups (Figures 3C–G).

### Pinch1/2 loss does not exacerbate surgically induced OA lesions in mice

**FIGURE 4:**
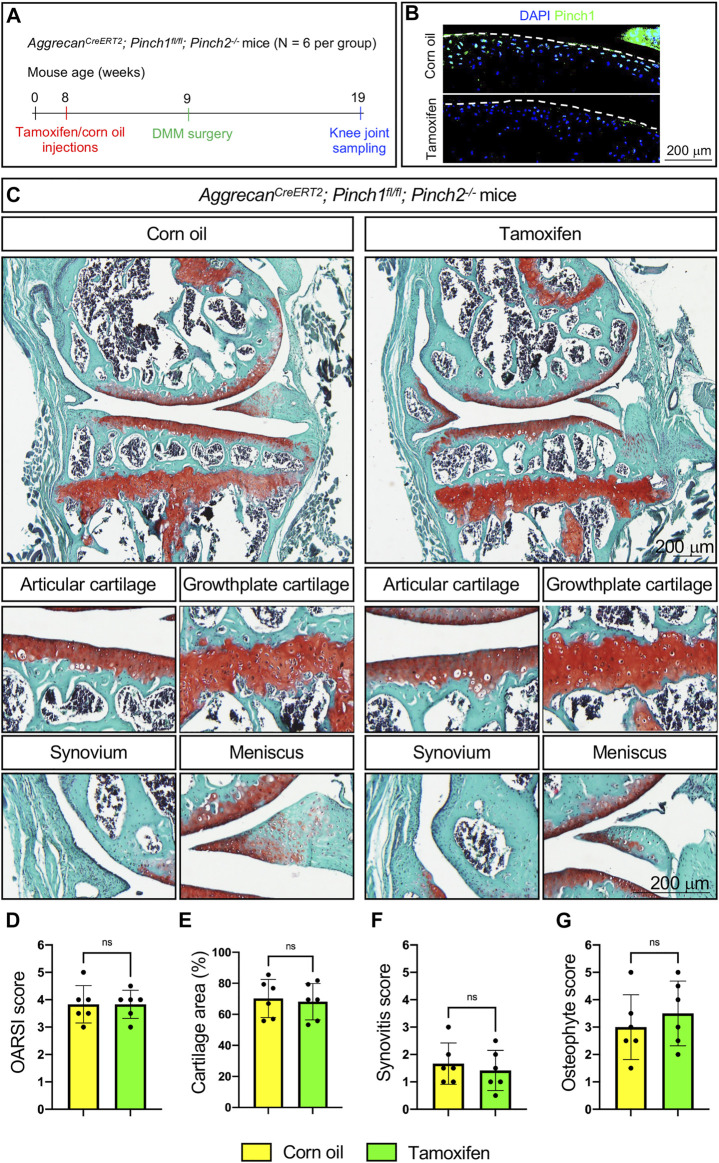
Pinch1/2 loss does not exacerbate surgically induced OA lesions in mice. **(A)** A schema illustrating the experimental design. **(B)** Immunofluorescence staining of Pinch1 in articular cartilage. Scale bar: 200 μm. **(C)** Representative safranin O and fast green-stained images of knee joint sections from tamoxifen- or corn oil-treated Aggrecan^CreERT2^; Pinch1^flox/flox^; Pinch2^−/−^ mice at 8 weeks after DMM surgery. Higher magnification images in lower panels are showing the areas of articular cartilage, growth-plate cartilage, synovium, and meniscus. Scale bar: 200 μm **(D–G)** Quantitative histological analyses of OARSI score, cartilage areas, synovitis score, and osteophyte score using knee joint sections. Note: N = 6 mice for each group. Results are expressed as mean ± standard deviation (s.d.). ns: not significant.

Next, we investigated whether Pinch1/2 deficiency could exacerbate the OA lesions in a destabilization of the medial meniscus (DMM) mouse model of OA. Six 2-month-old male Aggrecan^CreERT2^; Pinch1^flox/flox^; Pinch2^−/−^ mice were treated with tamoxifen, and another six age-matched Aggrecan^CreERT2^; Pinch1^flox/flox^; Pinch2^−/−^ mice were treated with corn oil. One week later, all mice were subjected to DMM surgery. Ten weeks after DMM surgery, all mice were sacrificed, and the knee joint samples were collected (Figure 4A). The Pinch1 expression was markedly decreased in the articular cartilage of tamoxifen-treated mice, as compared with control mice (Figure 2B). Histological analyses showed that DMM surgery successfully induced multiple OA lesions, including cartilage degradation and osteophyte formation (Figure 4C); nonetheless, the severity of OA lesions was comparable between tamoxifen- and corn oil-treated groups (Figures 4D–G).

## Methods

### Animal

The generation of Pinch1^flox/flox^ and Pinch2^−/−^ mice was previously described (Lei et al., 2020). To obtain the Aggrecan^CreERT2^; Pinch1^flox/flox^; Pinch2^−/−^ mice, Pinch1^flox/flox^ mice were first crossed with Pinch2^−/−^ mice to generate Pinch1^flox/+^; Pinch2^+/−^ mice. The Pinch1^flox/+^; Pinch2^+/−^ mice were then bred with each other to generate Pinch1^flox/flox^; Pinch2^−/−^ mice. Meanwhile, the Pinch1^flox/flox^ mice were crossed with the Aggrecan^CreERT2^ mice to generate Aggrecan^CreERT2^; Pinch1^flox/+^ mice. The Aggrecan^CreERT2^; Pinch1^flox/+^ mice were crossed with each other to obtain Aggrecan^CreERT2^; Pinch1^flox/flox^ mice. Finally, the Aggrecan^CreERT2^; Pinch1^flox/flox^ mice were bred with the Pinch1^flox/flox^; Pinch2^−/−^ mice to generate the Aggrecan^CreERT2^; Pinch1^flox/flox^; Pinch2^+/−^ mice. Then the Aggrecan^CreERT2^; Pinch1^flox/flox^; Pinch2^+/−^ mice were bred with each other to generate Aggrecan^CreERT2^; Pinch1^flox/flox^; Pinch2^−/−^ mice. It should be noted that the Pinch2 gene is globally inactivated in the Aggrecan^CreERT2^; Pinch1^flox/flox^; Pinch2^−/−^ mice. For inducible deletion of the Pinch1 gene in aggrecan-expressing chondrocytes, 2-month-old male Aggrecan^CreERT2^; Pinch1^flox/flox^; Pinch2^−/−^ mice were administrated with tamoxifen (Sigma T5648, 100 mg/kg per body weight/day) *via* intraperitoneal injections as previously described (Wu et al., 2022a; Wu et al., 2022b; Chen et al., 2022). Age-matched male Aggrecan^CreERT2^; Pinch1^flox/flox^; Pinch2^−/−^ mice were administrated with corn oil and used as the control group. All research protocols in this study were approved by the Institutional Animal Care and Use Committees (IACUC) of the Southern University of Science and Technology.

### Histology and immunofluorescent analyses

The histological analyses of mouse knee joints were performed according to our previously established protocols (Liu et al., 2021; Wu et al., 2022a; Wu et al., 2022b; Lai et al., 2022). The 5-μm thick knee joint sections were stained with safranin O and fast green using a commercial kit (Solarbio, Cat#G1371). The OA-related parameters, including the OARSI score, synovitis score, and osteophyte score were quantified using the histological scoring systems as previously described (Wu et al., 2022a). The areas of safranin O-stained articular cartilage were measured by ImageJ (version 1.53k) as previously described (Wu et al., 2022a). Representative images of safranin O and fast green staining were selected based on the mean values of histological scores. Immunofluorescent (IF) staining was performed using antibodies against Pinch1 (Abcam, ab108609) and Pinch2 (Abcam, ab173008) according to our previously established protocol (Wang et al., 2019; Gao et al., 2021; Qu et al., 2022).

### Statistical analysis

All mice used in this study were randomly assigned to each group. Statistical analyses were completed using the Prism GraphPad. Results were expressed as mean ± standard deviation (s.d.). Two-tailed unpaired Student’s t-test was employed to compare the differences between the two groups. Differences with *p* < 0.05 were considered statistically significant.

## Discussion

Chondrogenesis is a unique process in which mesenchymal progenitor cells condense and differentiate into chondrocytes to form cartilages (Goldring, 2012). During development, cartilage is a transient and highly dynamic structure, which is gradually replaced by bone to enable skeletal growth. In contrast, adult articular cartilage is a more stable tissue that provides lifelong structural and functional integrity of synovial joints (Haseeb et al., 2021). We have previously reported that Pinch proteins play crucial roles in mediating chondrogenesis and cartilage formation during skeletal development (Lei et al., 2020). Pinch loss in Prx1-positive mesenchymal progenitor cells (Prx1^Cre^; Pinch1^flox/flox^; Pinch2^−/−^) severely impairs chondrogenesis, leading to chondrocyte death and limb shortening in mice. These findings prompted us to test whether Pinch proteins have a role in maintaining the homeostasis of adult cartilage and whether alteration in their expression is associated with OA. Unexpectedly, results from this study demonstrate that deleting Pinch expression in aggrecan-expressing chondrocytes (Aggrecan^CreERT2^; Pinch1^flox/flox^; Pinch2^−/−^) has no effects on articular cartilage structure in adult mice. Besides articular cartilage, other aggrecan-expressing joint tissues, including growth plate cartilage and meniscus, were all comparable between Pinch-deficient mice and control mice. Moreover, we have tested whether Pinch loss could affect the cartilage structure and OA progression in both aging- and DMM-induced OA mouse models. The results showed no significant difference in OA-related histological parameters between Pinch-deficient mice and control mice in both spontaneous and surgically-induced OA models. These results, along with results from our previous study, reveal the distinct roles of Pinch proteins in cartilage tissues during development and adulthood.

The IPP complex, comprising ILK, Pinch, and parvin, is an essential component of the FA complex (Stiegler et al., 2013). It stabilizes the connection between the actin cytoskeleton and the cytoplasmic tail of *β* integrins, whereas loss of the IPP complex significantly impairs the FA formation (Li et al., 1999; Vakaloglou Katerina et al., 2016). The assembly of the IPP complex is crucial for the stability and cellular function of its individual protein component (Zhang et al., 2002; Stiegler et al., 2013). Loss of either ILK or Pinch results in the degradation of the other two protein components of the IPP complex (Fukuda et al., 2003; Li et al., 2005). Upon integrin activation, Kindlin-2 directly binds to *β* integrin tails and interacts with ILK for the recruitment of the IPP complex to the FA site (Montanez et al., 2008; Qadota et al., 2012). Loss of Kindlin-2 results in the absence of ILK or Pinch in the FA complex (Wickström et al., 2010). Our previous studies have shown that both Kindlin-2 and Pinch proteins are required for chondrogenesis and skeletal development in mice (Wu et al., 2015; Lei et al., 2020). Most recently, we have demonstrated that the expression of Kindlin-2 is also critical for the maintenance of cartilage homeostasis during adulthood (Wu et al., 2022a; Lai et al., 2022). It is interesting to compare the phenotypes of mice lacking Kindlin-2 in adult cartilage (Aggrecan^CreERT2^; Kindlin-2^flox/flox^) in our previous study with mice lacking Pinch1/2 in adult cartilage (Aggrecan^CreERT2^; Pinch1^flox/flox^; Pinch2^−/−^) in this study. Loss of Kindlin-2 causes multiple severe spontaneous OA-like phenotypes, including the progressive degradation of articular cartilage, synovitis, and osteophyte formation, whereas loss of Pinch proteins causes no obvious abnormalities. These results suggest that Kindlin-2 plays more critical roles than the Pinch proteins in aggrecan-expressing chondrocytes for the maintenance of adult cartilage homeostasis.

In summary, these results suggest that, although Pinch proteins are essential for chondrogenesis and cartilage development, their expression seems not to be indispensable for adult cartilage. Whether other FA-related proteins, such as Talin, Vinculin, and Paxillin, are involved in cartilage development and homeostasis warrants further investigations in future studies.

## Data Availability

The raw data supporting the conclusions of this article will be made available by the authors, without undue reservation.
